# Construction of high resolution genetic linkage maps to improve the soybean genome sequence assembly Glyma1.01

**DOI:** 10.1186/s12864-015-2344-0

**Published:** 2016-01-06

**Authors:** Qijian Song, Jerry Jenkins, Gaofeng Jia, David L. Hyten, Vince Pantalone, Scott A. Jackson, Jeremy Schmutz, Perry B. Cregan

**Affiliations:** USDA-ARS, Soybean Genomics and Improvement Lab, Beltsville, MD 20705 USA; HudsonAlpha Institute for Biotechnology, Huntsville, Alabama 35806 USA; Department of Agronomy & Horticulture, Center for Plant Science Innovation, 322 Keim Hall, University of Nebraska, Lincoln, NE 68583 USA; Department of Plant Sciences, 2431 Joe Johnson Dr., University of Tennessee, Knoxville, TN 37996-4561 USA; Center for Applied Genetic Technologies, University of Georgia, Athens, GA 30602-6810 USA; Joint Genome Institute, 2800 Mitchell Drive, Walnut Creek, California 94598 USA

**Keywords:** Soybean, Wm82.a2.v1 assembly, BARCSOYSSR_1.0 database, SoySNP50K BeadChip, euchromatic and heterochromatic regions, linkage map

## Abstract

**Background:**

A landmark in soybean research, Glyma1.01, the first whole genome sequence of variety Williams 82 (*Glycine max* L. Merr.) was completed in 2010 and is widely used. However, because the assembly was primarily built based on the linkage maps constructed with a limited number of markers and recombinant inbred lines (RILs), the assembled sequence, especially in some genomic regions with sparse numbers of anchoring markers, needs to be improved. Molecular markers are being used by researchers in the soybean community, however, with the updating of the Glyma1.01 build based on the high-resolution linkage maps resulting from this research, the genome positions of these markers need to be mapped.

**Results:**

Two high density genetic linkage maps were constructed based on 21,478 single nucleotide polymorphism loci mapped in the Williams 82 x *G. soja* (Sieb. & Zucc.) PI479752 population with 1083 RILs and 11,922 loci mapped in the Essex x Williams 82 population with 922 RILs. There were 37 regions or single markers where marker order in the two populations was in agreement but was not consistent with the physical position in the Glyma1.01 build. In addition, 28 previously unanchored scaffolds were positioned. Map data were used to identify false joins in the Glyma1.01 assembly and the corresponding scaffolds were broken and reassembled to the new assembly, Wm82.a2.v1. Based upon the plots of the genetic on physical distance of the loci, the euchromatic and heterochromatic regions along each chromosome in the new assembly were delimited. Genomic positions of the commonly used markers contained in BARCSOYSSR_1.0 database and the SoySNP50K BeadChip were updated based upon the Wm82.a2.v1 assembly.

**Conclusions:**

The information will facilitate the study of recombination hot spots in the soybean genome, identification of genes or quantitative trait loci controlling yield, seed quality and resistance to biotic or abiotic stresses as well as other genetic or genomic research.

**Electronic supplementary material:**

The online version of this article (doi:10.1186/s12864-015-2344-0) contains supplementary material, which is available to authorized users.

## Background

As a tool for genetic research and breeding, genetic linkage maps have been widely used to discover the position and to clone genes controlling biotic and abiotic stress resistance, agronomic and seed quality traits and to facilitate marker-assisted selection of the traits with low heritability and/or high phenotyping cost. In soybean, the first molecular genetic linkage map was reported in 1990 [1]. The map contained 150 restriction fragment length polymorphism (RFLP) markers that were mapped using an F_2_ population with 60 progeny derived from a cross of A81-356022 (*G. max*) × PI468916 (*G. soja*). Subsequently, a map with 130 RFLPs was constructed based on an F_2_ population with 69 progeny from a cross of Minsoy × Noir 1 [2], and a map with 165 RFLPs, 25 random amplified polymorphic DNA (RAPD) markers and 650 amplified fragment length polymorphisms (AFLPs) based on 300 recombinant inbred lines (RILs) from PI437654 × BSR101 [3] were reported. The early genetic linkage maps were primarily based on RFLP or AFLP markers and due to the lack of polymorphism or the complexity of the multiple banding patterns with these markers, simple sequence repeat (SSR) or microsatellite markers were proposed and then evaluated for the construction of genetic linkage maps [4, 5]. Cregan et al. (1999) [6] developed three separate linkage maps containing a total of 1421 markers including 606 SSRs, 689 RFLPs, 79 RAPDs and 47 other markers. These markers were mapped using three RIL populations: the Minsoy × Noir 1 population with 240 RILs, the A81-356022 × PI468916 population with 57 F2 plants, and the Clark × Harosoy population with 59 F_2_ plants and resulted in 20 linkage groups which were assumed to correspond to the 20 pairs of soybean chromosomes. Song et al. (2004) [7] constructed an integrated soybean linkage map using the three mapping populations used by Cregan et al. (1999) [6] as well as two additional mapping populations from Minsoy × Archer with 233 RILs, and Archer × Noir 1 with 240 RILs. The consensus map contained 1849 markers including 1015 SSRs, 709 RFLPs, 73 RAPDs and 52 other markers [7]. As large numbers of expressed sequence tags (ESTs) and genomic sequence became available in later years, Choi et al. (2007) [8] discovered >5500 single nucleotide polymorphism (SNP) markers by comparing DNA sequences acquired from a set of diverse genotypes after PCR amplification and sequence analysis of the EST or genomic sequences. A total of 1141 of the 5500 SNPs were mapped using three mapping populations including the Minsoy × Noir 1 with 164 RILs, Minsoy × Archer with 89 RILs as well as the Evans × PI 209332 with75 RILs [8]. Hyten et al. (2010) [9] added 2651 additional SNPs to the linkage maps created by Choi et al. (2007) [8] using the same Minsoy × Noir 1, Minsoy × Archer and Evans × Peking populations [9]. All of the molecular markers on these linkage maps were developed before the soybean whole genome sequence was available, thus, the markers were not evenly distributed and did not sufficiently cover all of the genomic regions of the soybean genome with a total sequence length >1100 Mb [10].

The Williams 82 Glyma1.01 whole genome sequence was completed and published in 2010 [11]. The genome sequence is widely used for the study of gene structure [12–14], syntenic relationships among legume species [15–18], identification of genes [19–22], the development of additional molecular markers and for other uses. Song et al. (2013) [23] identified 209,903 SNPs by mapping short reads from each of eight soybean accessions which included six cultivated (*Glycine max* (L.) Merr.) and two wild soybean (*G. soja* Sieb. & Zucc.) genotypes and selected 60,800 SNPs for the design of the SoySNP50K Illumina Infinium BeadChip [23]. The BeadChip has been successfully used to genotype the entire USDA Soybean Germplasm Collection containing 19,652 accessions including 1168 wild and 18,484 cultivated soybean accessions [24], the dataset is available at Soybase, the USDA, ARS Soybean Genetics and Genomics Database, http://www.soybase.org/snps/download.php) and is being used for genome-wide association analysis [25–29], quantitative trait loci (QTL) analysis [26], genetic diversity analysis and the identification of regions associated with domestication and selection imposed by modern breeding. In addition, Song et al. (2010) [30] identified a total of 210,990 SSRs with di-, tri-, and tetranucleotide repeats of five or more in the soybean whole genome sequence which included 61,458 SSRs consisting of repeat units of di- (≥10), tri- (≥8), and tetranucleotide (≥7), and developed a database (BARCSOYSSR_1.0) of locus-specific SSR markers with a high likelihood of polymorphism. A database with the primer sequences and their genome positions for 33,065 SSRs in the Glyma1.01 assembly was created [30]. The database also included the physical positions of 3322 SNPs in the Glyma1.01 build, which were mapped by Hyten et al. (2010) [9]. These molecular markers plus the markers developed previously [6–9, 31–35] are being used by researchers in the soybean community. However, with the updating of the Glyma1.01 build based on the high-resolution linkage maps resulting from this research, the genome positions of these markers need to be redefined.

The Glyma1.01 build captured approximately 975 Mb of sequence across the 20 chromosomes. The Glyma1.01 whole genome sequence contained 236 unanchored scaffolds with lengths ranging from 10 to 100 kb and 51 unanchored scaffolds with lengths greater than 100 kb. The assembly was basically built based on the integrated linkage maps [7, 9] and a genetic map with additional markers specifically selected to aid in the pseudomolecule assembly [36]. However, the marker density on any one of these linkage maps was still insufficient to fully cover all regions of the soybean genome. In addition, the number of RILs genotyped for the construction of the previous linkage maps was relatively small (60–240 RILs) [7, 9]. These deficiencies may result in low resolution, large gaps, and incorrect marker order in the linkage maps, and in turn, may cause incorrect orientation or misplacement of scaffolds in the Glyma1.0 whole genome sequence assembly of soybean.

The objectives of this research were to construct high resolution linkage maps using large mapping populations, to identify misplaced or incorrectly orientated genomic regions, to anchor additional scaffolds in the Glyma1.01 assembly, and to position SSR and SNP markers in the Wm82.a2.v1assembly.

## Results

### Construction of high resolution linkage maps

A total of 23,814 SNPs were polymorphic among 1083 RILs in the Williams 82 x PI479752 (WP) and 17,150 SNPs among the 922 RILs in the Essex x Williams 82 (EW) population. After elimination of SNPs with missing >10 % or segregation distortion significant at the 1 % level based on *χ*^2^ tests, 21,478, 11,922 and 27,431 SNPs were mapped in the WP, EW and WP + EW populations, respectively. The number of mapped SNPs in each linkage group ranged from 825 to 1910 in the WP, 132–1313 in the EW and 938–2481 in the WP + EW populations. The total genetic linkage map distance was 2445.8 cM in the WP and 2647.6 cM in the EW population (Table 1 and Additional file 1: Table S1).

**Table 1:** Number of SNPs mapped to each linkage group, linkage group length based on the Williams 82 × PI479752 (WP) and the Essex × Williams 82 (EW) populations and the number of SNPs common to the two populations and SNPs unique to one population

Linkage Group	WP		EW		Number of SNPs common to the WP and EW populations	Number of unique SNPs
	Number of SNPs	Length (cM)	Number of SNPs	Length (cM)		
Gm01	591	107.9	486	121.4	115	962
Gm02	1325	132.2	821	168.1	468	1678
Gm03	842	113.3	472	128.4	203	1111
Gm04	1185	112.1	616	143.2	349	1452
Gm05	1128	121.5	507	127.4	293	1342
Gm06	980	162.0	629	158.8	256	1353
Gm07	1159	136.2	376	146.1	223	1312
Gm08	965	177.7	636	128.3	107	1494
Gm09	997	134.1	736	132.0	374	1359
Gm10	1207	144.0	733	145.9	390	1550
Gm11	825	97.8	262	146.2	149	938
Gm12	938	106.6	383	127.0	213	1108
Gm13	1458	131.5	737	153.3	371	1824
Gm14	873	102.8	132	70.1	67	938
Gm15	1309	110.8	923	131.2	523	1709
Gm16	954	98.1	392	99.5	208	1138
Gm17	1037	129.3	490	115.2	284	1243
Gm18	1910	107.2	1313	126.4	742	2481
Gm19	953	109.0	936	125.5	414	1475
Gm20	842	111.7	342	153.6	220	964
Total	21478	2445.8	11922	2647.6	5969	27431

### Identification of misassembled genomic regions or anchorable scaffolds in Glyma1.01

Of the 21,478 SNPs mapped in WP and 11,922 in EW, 5969 SNPs were present in both populations and the number of common SNPs per chromosome ranged from 67 on chromosome Gm14 to 742 on chromosome Gm18. Marker order on the genetic linkage maps was used to identify major genomic regions of the Glyma1.01 that required reorientation and/or re-positioning. Analysis showed that the order of the common markers on the two linkage maps was highly consistent and the order of the SNPs was generally consistent with their physical positions along the corresponding chromosomes of Glyma1.01 (Additional file 2: Figure S1). However, there were 22 regions or single markers that required re-positioning or reorientation based upon marker orders supported by both the WP and EW mapping data (Table 2). In these regions, the SNP markers had consistent order along the linkage maps in both mapping populations but their order was not consistent with physical position in Glyma1.01. For example, there were regions on Gm04, Gm05, Gm10 and Gm13 where the order of SNPs on both linkage maps was identical, but the order of those SNPs in the Glyma1.01 assembly was reversed (Additional file 2: Figure S1). In addition, a number of individual markers or sets of markers identified sequence that was placed on the wrong chromosome (Table 2). There were a total of 15 regions that required re-positioning or reorientation based upon marker orders available from either the WP or EW mapping data (Table 2). In addition, 28 unanchored scaffolds with a total length of 3.6 Mb in Glyma1.01 were anchored as a result of markers in either the WP or EW map, or both, that defined the scaffold genome position (Table 3).

**Table 2:** Regions or single markers in Glyma1.01 that required re-positioning or reorientation based upon marker orders supported by both or either the W82 x PI79752 and/or the Essex x W82 mapping data

First marker in the interval			Last marker in the interval			Comment	Supporting map data
SNP ID	Chromosome	Physical position	SNP ID	Chromosome	Physical position		
BARC_1.01_Gm02_22523407_T_C	Gm02	22,523,407	BARC_1.01_Gm02_22917212_A_G	Gm02	22,917,212	Move to Gm15	WP and EW
BARC_1.01_Gm02_26182810_A_G	Gm02	26,182,810	BARC_1.01_Gm02_27329992_A_G	Gm02	27,329,992	Move to Gm13	WP and EW
BARC_1.01_Gm02_43000450_C_T	Gm02	43,000,450	BARC_1.01_Gm02_43043202_A_C	Gm02	43,043,202	Re-orient	WP and EW
BARC_1.01_Gm03_5530681_A_G	Gm03	5530681	BARC_1.01_Gm03_6597027_G_A	Gm03	6597027	Re-orient	WP and EW
BARC_1.01_Gm04_29510350_C_A	Gm04	29,510,350	BARC_1.01_Gm04_29566738_A_G	Gm04	29,566,738	Re-orient and move to Gm18	WP and EW
BARC_1.01_Gm05_8031928_A_C	Gm05	8,031,928	BARC_1.01_Gm05_9066302_T_C	Gm05	9,066,302	Move to top of chromosome	WP and EW
BARC_1.01_Gm05_9616597_C_T	Gm05	9,616,597	BARC_1.01_Gm05_16324558_C_T	Gm05	16,324,558	Re-orient	WP and EW
BARC_1.01_Gm05_16504006_C_T	Gm05	16504006	BARC_1.01_Gm05_20385655_G_T	Gm05	20385655	Re-orient	WP and EW
BARC_1.01_Gm05_38634602_T_C	Gm05	38,634,602	BARC_1.01_Gm05_41919487_G_T	Gm05	41,919,487	Re-orient	WP and EW
BARC_1.01_Gm07_10457480_C_A	Gm07	10,457,480	BARC_1.01_Gm07_14773717_G_T	Gm07	14,773,717	Re-orient	WP and EW
BARC_1.01_Gm09_37436031_A_C	Gm09	37,436,031	BARC_1.01_Gm09_37478410_A_G	Gm09	37,478,410	Re-orient	WP and EW
BARC_1.01_Gm10_14435077_T_C	Gm10	14,435,077	BARC_1.01_Gm10_27968025_A_C	Gm10	27,968,025	Re-orient	WP and EW
BARC_1.01_Gm11_37808033_A_G	Gm11	37,808,033	BARC_1.01_Gm11_39163663_A_G	Gm11	39,163,663	Re-orient	WP and EW
BARC_1.01_Gm12_18007551_G_T	Gm12	18,007,551	BARC_1.01_Gm12_18239449_G_A	Gm12	18,239,449	Move to Gm04	WP and EW
BARC_1.01_Gm13_5491_A_G	Gm13	5,491	BARC_1.01_Gm13_20223181_A_G	Gm13	20,223,181	Re-orient	WP and EW
BARC_1.01_Gm13_35242360_T_C	Gm13	35,242,360	BARC_1.01_Gm13_35307167_A_G	Gm13	35,307,167	Move to Gm09	WP and EW
BARC_1.01_Gm15_10351491_G_T	Gm15	10,351,491	BARC_1.01_Gm15_10427384_A_G	Gm15	10,427,384	Move and Re-orient	WP and EW
BARC_1.01_Gm15_36006344_T_C	Gm15	36,006,344	BARC_1.01_Gm15_38303424_T_C	Gm15	38,303,424	Move and Re-orient	WP and EW
BARC_1.01_Gm17_9749711_A_G	Gm17	9,749,711				Move to Gm10	WP and EW
BARC_1.01_Gm18_24754213_G_T	Gm18	24,754,213	BARC_1.01_Gm18_27432506_A_G	Gm18	27,432,506	Move to Gm04	WP and EW
BARC_1.01_Gm19_12811558_G_A	Gm19	12811558	BARC_1.01_Gm19_17460363_C_A	Gm19	17460363	Re-orient	WP and EW
BARC_1.01_Gm20_10352781_A_G	Gm20	10,352,781	BARC_1.01_Gm20_19781743_T_C	Gm20	19,781,743	A number of changes needed	WP and EW
BARC_1.01_Gm01_16580419_T_G	Gm01	16580419	BARC_1.01_Gm01_17671586_G_A	Gm01	17671586	Re-orient	WP
BARC_1.01_Gm02_27407299_A_G	Gm02	27407299	BARC_1.01_Gm02_29498377_C_T	Gm02	29498377	Re-orient	WP
BARC_1.01_Gm03_16199297_C_T	Gm03	16199297	BARC_1.01_Gm03_22901336_G_T	Gm03	22901336	Re-orient	WP
BARC_1.01_Gm04_34743951_T_C	Gm04	34743951	BARC_1.01_Gm04_33785067_T_C	Gm04	33785067	Move to Gm20	WP
BARC_1.01_Gm05_30871172_T_C	Gm05	30871172	BARC_1.01_Gm05_30910003_G_A	Gm05	30910003	Move to Gm11	WP
BARC_1.01_Gm08_44242727_C_T	Gm08	44242727	BARC_1.01_Gm08_44632488_A_G	Gm08	44632488	Re-orient	WP
BARC_1.01_Gm10_42894189_C_T	Gm10	42894189	BARC_1.01_Gm10_43004105_A_C	Gm10	43004105	Re-orient	WP
BARC_1.01_Gm13_34645498_A_G	Gm13	34645498	BARC_1.01_Gm13_34658945_C_A	Gm13	34658945	Re-orient	EW
BARC_1.01_Gm14_48713607_A_G	Gm14	48713607	BARC_1.01_Gm14_48755126_G_A	Gm14	48755126	Re-orient	WP
BARC_1.01_Gm15_25823658_T_C	Gm15	25823658	BARC_1.01_Gm15_36378430_A_G	Gm15	36378430	Re-orient	EW
BARC_1.01_Gm16_5856598_G_A	Gm16	5856598	BARC_1.01_Gm16_5887676_G_A	Gm16	5887676	Re-orient	WP
BARC_1.01_Gm16_17407537_T_G	Gm16	17407537	BARC_1.01_Gm16_22593496_G_A	Gm16	22593496	Re-orient	WP
BARC_1.01_Gm19_3021_T_C	Gm19	3021	BARC_1.01_Gm19_567731_A_G	Gm19	567731	Re-orient	WP
BARC_1.01_Gm20_7082863_T_G	Gm20	7082863	BARC_1.01_Gm20_7419439_G_A	Gm20	7419439	Re-orient	WP
BARC_1.01_Gm20_18531300_T_C	Gm20	18531300	BARC_1.01_Gm20_7419439_G_A	Gm20	20977430	Re-orient	WP

**Table 3:** Twenty-eight previously unanchored scaffolds for which there were markers in either the Williams 82 × PI479752 (WP) or the Essex × Williams 82 (EW) map, or both, to define their genome position

SNP ID	Scaffold	Physical Position of SNP in the scaffold	LG based on WP population	Position based on WP population (cM)	LG_based on EW population	Position based on EW population (cM)	Total scaffold length
BARC_1.01_scaffold_1036_3469_T_C	scaffold_1036	3469	8	117.851			5173
BARC_1.01_scaffold_1448_1683_A_C	scaffold_1448	1683	17	80.252			3995
BARC_1.01_scaffold_1448_67_G_A	scaffold_1448	67	17	80.252			
BARC_1.01_scaffold_1454_1730_G_A	scaffold_1454	1730	1			87.464	3982
BARC_1.01_scaffold_1484_809_T_C	scaffold_1484	809	10	57.485			4368
BARC_1.01_scaffold_1605_1791_T_C	scaffold_1605	1791	9	82.196	9	80.192	3584
BARC_1.01_scaffold_169_21520_G_A	scaffold_169	21520	11	90.373			25752
BARC_1.01_scaffold_169_9083_G_T	scaffold_169	9083	11	90.373			
BARC_1.01_scaffold_2048_399_C_A	scaffold_2048	399	16			69.671	1779
BARC_1.01_scaffold_2182_1012_T_C	scaffold_2182	1012	13	92.688	13	134.477	1349
BARC_1.01_scaffold_22_540761_G_A	scaffold_22	540761	8			120.532	1088050
BARC_1.01_scaffold_22_985719_G_A	scaffold_22	985719	8			120.532	
BARC_1.01_scaffold_2280_754_G_A	scaffold_2280	754	18			69.307	1018
BARC_1.01_scaffold_23_881897_T_C	scaffold_23	881897	1	46.789			939397
BARC_1.01_scaffold_24_197620_T_C	scaffold_24	197620	10			52.909	634454
BARC_1.01_scaffold_245_10767_A_G	scaffold_245	10767	11	87.989			17525
BARC_1.01_scaffold_248_8179_A_G	scaffold_248	8179	7	12.253			17311
BARC_1.01_scaffold_303_12268_T_G	scaffold_303	12268	3	28.82			17325
BARC_1.01_scaffold_317_4132_A_G	scaffold_317	4132	9	101.035			14271
BARC_1.01_scaffold_36_219042_G_A	scaffold_36	219042	2	75.178	2	97.615	280716
BARC_1.01_scaffold_469_2885_C_T	scaffold_469	2885	1			55.543	10200
BARC_1.01_scaffold_476_2115_T_C	scaffold_476	2115	9	57.081	9	48.989	10120
BARC_1.01_scaffold_476_9307_A_C	scaffold_476	9307	9	57.118	9	49.034	
BARC_1.01_scaffold_48_40550_T_C	scaffold_48	40550	9	134.091			139886
BARC_1.01_scaffold_554_3651_G_A	scaffold_554	3651	16			99.322	9124
BARC_1.01_scaffold_66_159931_A_G	scaffold_66	159931	9	60.405	9	54.911	170827
BARC_1.01_scaffold_732_107_A_G	scaffold_732	107	18	63.953			6997
BARC_1.01_scaffold_825_3928_G_T	scaffold_825	3928	2	75.353			6293
BARC_1.01_scaffold_84_64248_T_C	scaffold_84	64248	10	52.937			69299
BARC_1.01_scaffold_91_31407_G_A	scaffold_91	31407	9	58.919	9	51.365	63120
BARC_1.01_scaffold_938_1798_A_G	scaffold_938	1798	15	83.907	15	102.19	6175
BARC_1.01_scaffold_97_54858_G_A	scaffold_97	54858	9	58.919			57671
Total length of newly anchored scaffolds							3609761

### The Wm82.a2.v1 assembly

Based on the two dense linkage maps and additional analyses, sequence breaks from Glyma1.01 were identified and reassembled. The new build of the 20 chromosomes captured 949.2 Mb. The total sequence including the 1170 unmapped scaffolds was 978.5 Mb. The plots of the genetic on physical distance of the SNPs in Glyma1.01 and Wm82.a2.v1 showed that major regions, such as on Gm05 and Gm13 with an inconsistent order of SNPs on linkage maps vs. physical position in the Glyma1.01 build were corrected in the Wm82.a2.v1 assembly (Additional file 3: Figure S2). Further comparison of the physical positions of the SNPs in Glyma1.01 vs. Wm82.a2.v1 showed that sequence assembly errors in the regions indicated in Table 2 and Table 3 were all corrected in Wm82.a2.v1 (Additional file 1: Table S1). In addition, a total of 28 scaffolds with mapped SNP markers were anchored to the new build. The new assembly which is completed at the Department of Energy, Joint Genome Institute is available at http://www.phytozome.jgi.doe.gov/pz/portal.html.

Based upon the plots of genetic distance on physical distance of the SNPs in Wm82.a2.v1 and mapped in either the WP or EW population, the regions with high and low recombination rate were defined. These plots allowed the delimitation of the approximate positions of euchromatic and heterochromatic regions along each chromosome (Table 4). The two regions covered approximately 43 % and 47 % of the total estimated genome sequence, respectively.

**Table 4:** Approximate positions of heterochromatic and euchromatic regions in the Wm82.a2.v1 whole genome sequence

Chromosome	Heterochromatic region (Mb)	Euchromatic region (Mb)
Chr01	8.1-47.4	1-8.1; 47.4-56.8
Chr02	16.0-38.2	1-16.0; 38.2-48.6
Chr03	6.9-33.4	1-6.9; 33.4-45.8
Chr04	10.4-43.5	1-10.4; 43.5-52.4
Chr05	6.4-30.2	1-6.4; 30.2-42.2
Chr06	18.2-44.4	1-18.2; 44.4-51.4
Chr07	17.7-34.6	1-17.7; 34.6-44.6
Chr08	22.9-40.4	1-22.9; 40.4-47.8
Chr09	6.4-38.8	1-6.4; 38.8-50.2
Chr10	6.9-36.9	1-6.9; 36.9-51.5
Chr11	11.4-30.0	1-11.4; 30.0-34.7
Chr12	8.2-32.4	1-8.2; 32.4-40.0
Chr13	0-13.3	1-0; 13.3-45.8
Chr14	9.7-43.7	1-9.7; 43.7-49.0
Chr15	18.3-43.0	1-18.3; 43.0-51.7
Chr16	8.3-26.8	1-8.3; 26.8-37.8
Chr17	14.3-35.8	1-14.3; 35.8-41.6
Chr18	20.5-43.3	1-20.5; 43.3-58.0
Chr19	8.9-34.3	1-8.9; 34.3-50.7
Chr20	3.2-33.7	1-3.2; 33.7-47.9
Total	501.4 (53 %)	447.8 (47 %)

### Positions of commonly used markers in the Wm82.a2.v1 assembly

Of the 33,065 SSRs and 3322 SNPs in the BARCSOYSSR_1.0 database, 32,602 SSRs and 3314 SNPs were unambiguously positioned in the Wm82.a2.v1 assembly. A total of 2122 SNPs and 7092 SSRs were in the genes defined in Wm82.a2.v1 and the total number of unique genes in which these SSR and SNP markers resided was 7686 (Additional file 4: Table S2).

Among the 60,800 SNPs originally selected for inclusion in the SoySNP50K BeadChip [23], 60,556 SNPs were positioned in the new assembly and a total of 20,271 SNPs were in 14,880 different genes. The positions of 244 SNPs in the Wm82.a2.v1 assembly could not be determined (Additional file 1: Table S1).

## Discussion

The two linkage maps created in this study have the highest density of markers and are based on the largest number of recombinant inbred lines that have been reported in soybean to date. Simulation studies indicated that a low number of RILs in a population frequently caused inversions of marker order and breakage in linkage groups and that the precision of the maps is highly dependent on the number of RILs [37]. For the purpose of integrating large numbers of markers into a linkage map, the WP population which was derived from the cross of cultivated by wild soybean accessions was developed. The large genetic divergence between the two subspecies allowed us to identify and map large numbers of SNPs in a single population. One concern with the linkage maps from *G. max* x G. *soja* was the possibility of paracentric inversions and reciprocal translocations between the cultivated soybean and certain wild soybean accessions [38, 39]. However, we did not observe such regions in the linkage maps of WP based on the order of approximately 6000 common SNPs mapped in both the WP and EW populations.

Besides the number of markers and size of the RIL populations, utilization of evenly distributed markers across the whole soybean genome was also essential to ensure maps with high resolution. The SNPs in the SoySNP50K BeadChip were carefully selected in order to equalize the distance between selected SNPs in the euchromatic and heterochromatic regions along each chromosome and the BeadChip was able to generate high quality genotyping data [23]. The resulting two linkage maps had better coverage and higher resolution than any other soybean linkage maps reported previously. The high quality of the two linkage maps is supported by the very consistent order of the common markers in the two maps.

Even though the Glyma1.01 build was well constructed, we identified regions where the marker physical order was inconsistent with the WP and EW linkage maps. Most of these regions either had insufficient marker numbers or lacked markers with recombination in the previous linkage maps [7, 9, 36] on which the Glyma1.01 assembly was based. The misassembled or improperly oriented regions identified by our linkage maps covered all of the major regions reported by Lee et al. (2013) [40] and the regions were moved or re-assembled in the Wm82.a2.v1 assembly. Of course, refinement of some regions may still be required especially in the heterochromatic regions where limited recombination was observed.

In order to determine the approximate positions of the euchromatic and heterochromatic regions of the genome, the cumulative genetic distances (cM) were plotted against their corresponding cumulative physical distance (Mbp) via the mapped SNP loci positions on the genetic linkage map and their genome sequence position along each chromosome. The region between the two inflection points of the cumulative genetic distance against cumulative physical distance on the plot was defined as the heterochromatic region [23]. The reliability of defining heterochromatic regions using this method was validated by the conventional 4,6-diamidino-2-phenylindole dihydrochloride staining method in rice [41].

Because of the many reports of genes/QTL in the soybean genome positioned using SSR or SNP markers, the corresponding physical position of the molecular markers in the new assembly vs. the older assembly is frequently requested by users. We identified physical positions for almost all of the markers in the BARCSOYSSR_1.01 database and the SoySNP50K BeadChip in the Wm82.a2.v1 vs. the Glyma1.01 assemblies. The updated information is anticipated to facilitate the identification of molecular markers in desired positions of the genome and make the SSR and SNP databases more user-friendly.

## Conclusions

Two high density genetic linkage maps of soybean based on 21,478 SNP loci mapped in the *G. max* x *G. soja* population with 1083 recombinant inbred lines and 11,922 SNP loci mapped in the *G. max* x *G. max* population with 922 RILs were constructed. The maps contained the highest number of markers and were constructed based on the largest mapping populations in soybean to date. With the high density genetic linkage maps, false joins or mis-placed scaffolds and unanchored scaffolds in the first version of the soybean whole-genome sequence assembly (Glyma1.01) were identified and the corresponding scaffolds were broken or reassembled to a new Wm82.a2.v1 assembly which is available at the site http://www.phytozome.jgi.doe.gov/pz/portal.html/ of the Department of Energy, Joint Genome Institute. In addition, the euchromatic and heterochromatic regions along each chromosome of the soybean were delimited and the positions of commonly used soybean SSR and SNP markers were determined based on the Wm82.a2.v1 assembly. The information will facilitate the genetic and genomics research in soybean.

## Methods

### Mapping populations

A cross between cultivated soybean (*Glycine max* L. Merr.) Williams 82 and wild soybean (*G. soja* Sieb. et Zucc.) PI479752 (WP) was made at Beltsville, MD. The WP population consists of 1083 F_5_-derived RILs. The Essex × Williams 82 population with 922 F_5_-derived RILs was developed at the University of Tennessee, Knoxville, TN. One of the parents in both mapping populations was Williams 82, which is the cultivar that was used in the synthesis of the first whole-genome sequence of the soybean provided in the Glyma1.01assembly [11].

### Genotyping RILs of the mapping populations with the SoySNP50K BeadChip

Song et al. (2013) [23] identified 209,903 SNPs by mapping short reads from each of eight soybean accessions which included six cultivated and two wild soybean genotypes and selected 60,800 SNPs for inclusion in an Illumina Infinium BeadChip that ultimately contained more than 52,000 SNPs. The SNPs for the SoySNP50K BeadChip were selected so as to equalize the distance between selected SNPs in the euchromatic and heterochromatic regions, increase assay success rate, and minimize the number of SNPs with low minor allele frequency. Of the 60,800 SNPs selected for the BeadChip, 50,701 were targeted to euchromatic regions and 10,000 to heterochromatic regions of the 20 soybean chromosomes. In addition, 99 SNPs were targeted to unanchored sequence scaffolds. The BeadChip was used to genotype the RILs in the WP and the EW populations using the Illumina platform following the Infinium® HD Assay Ultra Protocol (Illumina, Inc. San Diego, CA) and the SNP alleles were called using the GenomeStudio Genotyping Module v1.8.4 (Illumina, Inc. San Diego, CA) as described previously [23].

### Construction of the high-density linkage maps

Linkage maps for the WP and EW populations were created using the MSTMap software [42] and the genetic distance between SNPs was calculated using JoinMap 4.0 [43]. Before linkage map analysis, loci with segregation distortion in the population (p < 0.01) or with missing data >10 % were eliminated. RILs with missing data >10 % were also removed. In order to reduce the time required to determine the order and genetic distance of the SNPs in each linkage group, SNPs with identical allele segregation patterns among RILs of WP or EW populations were clustered into groups, and only one SNP from each group was included in linkage analysis. The remaining SNPs were assigned to the same linkage map position as the representative SNP after completion of the linkage analysis. A LOD of 11 was used to cluster the markers into linkage groups. Recombination values were converted to genetic distances using the Kosambi mapping function [43].

### Identification of genomic regions in the Glyma1.01 assembly that required re-positioning or reorientation

Genetic linkage map positions of SNPs on the linkage maps of WP and EW were compared with their physical positions in the Glyma1.01assembly and regions that required re-positioning or reorientation were identified based upon marker orders supported by the WP and/or the EW mapping data and scaffolds with false joins were broken and re-assembled. The physical positions of these SNPs in the Glyma1.01 were previously reported by Song et al. (2013) [23].

### Physical positions of commonly used SSR and SNP markers in the new assembly- Wm82.a2.v1

The BARCSOYSSR_1.0 database consists of 3322 SNPs and 33,065 SSRs [30], and the SoySNP50K BeadChip contained 52,041 SNPs selected from the soybean genome. In order to position these loci in the Wm82.a2.v1 soybean genome sequence, source sequences of the SSR and the sequences flanking the SNP loci were aligned to the Wm82.a2.v1 soybean sequences using standalone Megablast software (http://www.ncbi.nlm.nih.gov/blast/megablast.shtml) with W = 50, cutoff percentage of alignment = 99 and low complexity filtered. The primer sequences of the SSR loci were mapped to the genome sequence using the standalone software e-PCR (ftp://ftp.ncbi.nih.gov/pub/schuler/e-PCR/). SSR positions were definitively determined if both the source sequences and primer sequences of the SSRs aligned to the same region of the genome sequence with expected e-PCR amplicon length and with the SSR motif between the two primer sequences. High stringency alignment (gap = 0, number of mismatch = 0) with e-PCR of primer sequences to the genome sequence was used to map the primer sequences.

### Availability of supporting data

The SNP information is deposited in the dbSNP database of NCBI (ss715578401-ss715639200). The new soybean whole genome sequence assembly (Wm82.a2.v1) which is completed at the Department of Energy, Joint Genome Institute is available at http://www.phytozome.jgi.doe.gov/pz/portal.html/. The remaining data sets supporting the results of this article are included within the article and its four additional files.

## Additional files

Additional file 1: Table S1. NCBI ssID, SNP ID of SoySNP50K SNPs (Song et al. [23]), genome position Glyma1.01, corresponding genome position in the Wm82.a2.v1 assembly, gene IDs of SNPs in the Wm82.a2.v1 assembly and genetic linkage group and linkage position of the SNPs in the Williams 82 x PI479752 (WP) and Essex x Williams 82 (EW) populations (XLS 10149 kb) Additional file 2: Figure S1. Consensus diagram of physical order (left) of common SNPs in Glyma1.01 vs. their genetic linkage map order on the EW (middle) and the WP (right) maps. Common SNP loci are connected with red lines. (DOCX 2614 kb) Additional file 3: Figure S2. Plots of genetic vs. physical distance of SNPs. Figures Gm01-Gm20, and Chr01-Chr20 are the plots of genetic on physical distance based on Glyma1.01 and Wm82.a2.v1, respectively. Blue and red lines are based on the Williams 82 × PI479752 and the Essex × Williams 82 populations, respectively. (DOCX 1660 kb) Additional file 4: Table S2. Genomic position and corresponding gene IDs of the SSR and SNP markers in the Wm82.a2.v1 assembly, the SSR and SNP markers were from the BARCSOYSSR_1.0 database (Song et al. [30]). (XLS 8876 kb)

## Abbreviations

AFLP: amplified fragment length polymorphisms
EW: Essex x Williams 82 population
QTL: quantitative trait loci
RAPD: random amplified polymorphic DNA
RFLP: restriction fragment length polymorphism
RILs: recombinant inbred lines
SNP: single nucleotide polymorphism
SSR: simple sequence repeat
WP: Williams 82 x PI479752 population
