# Supplementary material for: Construction of high resolution genetic linkage maps to improve the soybean genome sequence assembly Glyma1.01
Source: BMC Genomics. 2016 Jan 6;17:33. doi: 10.1186/s12864-015-2344-0 (PMC4704267; doi:10.1186/s12864-015-2344-0)
Supplement: Additional file 2: Figure S1. — Consensus diagram of physical order (left) of common SNPs in Glyma1.01 vs. their genetic linkage map order on the EW (middle) and the WP (right) maps. Common SNP loci are connected with red lines. (DOCX 2614 kb) [file 12864_2015_2344_MOESM2_ESM.docx]

Gm01

Gm02

Gm03

Gm04

Gm05

Gm06

Gm07

Gm08

Gm09

Gm10

Gm11

Gm12

Gm13

Gm14

Gm15

Gm16

Gm17

Gm18

Gm19

Gm20

Additional file 2: Figure S1. Consensus diagram of physical order (left) of common SNPs in Glyma1.01 vs. their genetic linkage map order on the EW (middle) and the WP (right) maps. Common SNP loci are connected with red lines.
