# Supplementary material for: Construction of high resolution genetic linkage maps to improve the soybean genome sequence assembly Glyma1.01
Source: BMC Genomics. 2016 Jan 6;17:33. doi: 10.1186/s12864-015-2344-0 (PMC4704267; doi:10.1186/s12864-015-2344-0)
Supplement: Additional file 3: Figure S2. — Plots of genetic vs. physical distance of SNPs. Figures Gm01-Gm20, and Chr01-Chr20 are the plots of genetic on physical distance based on Glyma1.01 and Wm82.a2.v1, respectively. Blue and red lines are based on the Williams 82 × PI479752 and the Essex × Williams 82 populations, respectively. (DOCX 1660 kb) [file 12864_2015_2344_MOESM3_ESM.docx]

Additional file 3: Figure S2. Plots of genetic vs. physical distance of SNPs. Figures Gm01-Gm20, and Chr01-Chr20 are the plots of genetic on physical distance based on Glyma1.01 and Wm82.a2.v1, respectively. Blue and red lines are based on the Williams 82 × PI479752 and the Essex × Williams 82 populations, respectively.
